# Pathogenicity of *Nosema* sp. (Microsporidia) in the Diamondback Moth, *Plutella xylostella* (Lepidoptera: Plutellidae)

**DOI:** 10.1371/journal.pone.0062884

**Published:** 2013-05-13

**Authors:** Nadia Kermani, Zainal-Abidin Abu-hassan, Hamady Dieng, Noor Farehan Ismail, Mansour Attia, Idris Abd Ghani

**Affiliations:** 1 School of Environmental and Natural Resource Sciences, University Kebangsaan Malaysia, Bangi, Selangor, Malaysia; 2 Faculty of Medicine, University Technology MARA, Shah Alam, Selangor, Malaysia; 3 School of Biological Sciences, Universiti Sains Malaysia, Penang, Malaysia; Instituto de Salud Carlos III, Spain

## Abstract

Biological control using pathogenic microsporidia could be an alternative to chemical control of the diamondback moth (DBM) *Plutella xylostella* (Lepidoptera: Plutellidae). The microsporidium *Nosema bombycis* (NB) is one of the numerous pathogens that can be used in the Integrated Pest Management (IPM) of DBM. However, its pathogenicity or effectiveness can be influenced by various factors, particularly temperature. This study was therefore conducted to investigate the effect of temperature on NB infection of DBM larvae. Second-instar larvae at different doses (spore concentration: 0, 1×10^2^,1×10^3^,1×10^4^, and 1×10^5^) at 15°, 20°, 25°, 30° and 35°C and a relative humidity(RH) of 65% and light dark cycle (L:D) of 12∶12. Larval mortality was recorded at 24 h intervals until the larvae had either died or pupated. The results showed that the spore concentration had a significant negative effect on larval survival at all temperatures, although this effect was more pronounced (92%) at 35°C compared with that at 20 and 30°C (≃50%) and 25°C (26%). Histological observations showed that *Nosema* preferentially infected the adipose tissue and epithelial cells of the midgut, resulting in marked vacuolization of the cytoplasm. These findings suggest that *Nosema* damaged the midgut epithelial cells. Our results suggest that *Nosema* had a direct adverse effect on DBM, and could be utilized as an important biopesticide alternative to chemical insecticides in IPM.

## Introduction

The diamondback moth (DBM) *Plutella xylostella* L. (Lepidoptera: Plutellidae) causes considerable economic losses worldwide to brassicaceous crops, and occasionally to other crops. Control of this pest is usually achieved through the application of synthetic insecticides that is estimated to cost more than US$1 billion/annum to control. Management costs and crop losses caused by DBM account for US$4–US$5 billion [1]. The high cost, environmental contamination, development of resistance to chemicals, and pest resurgence [2], [3] associated with the current DBM control practices have encouraged the search for alternatives that are more environment friendly. Microbial control is an environmentally sound and valuable option to control this pest. In Malaysia, *Nosema bombycis* Negali is one of the several pathogens of DBM in the field [4]. DBM mortality is higher in younger instars (first and second instars) than in the older instars. Further, even at low concentrations, infection is remarkably higher for both larvae and pupae in highlands than in lowlands [4].

The effect of temperature on the biology of *Nosema* needs to be investigated because it is one of the most important ecological factors for the development of insect populations. Therefore, this study investigated the effects of *Nosema* spore concentration on the different stages of DBM reared at different temperatures. Establishing a correlation between temperature and pathogenicity of *Nosema* infection would be beneficial in determining whether *Nosema* can be applied as a DBM controlling agent. The optimal temperature at which this pathogen might be more effective in controlling the pest was studied. This information might help in determining whether this pathogen could be used in integrated pest management (IPM) and whether the amount of pesticide required could be reduced considering that *Nosema-*infected populations are more susceptible to the toxicity of the insecticides. In insects, the midgut is a dynamic tissue of the alimentary canal that acts as the route of digestion and allows absorption of digested food. Thus, we studied the effect of Nosema on this active organ. The results are also expected to provide useful information on the histopathology effects on larvae caused by *Nosema*.

## Materials and Methods

### Diamondback Moth

Disease-free DBM larvae of the University Putra Malaysia strain were obtained from the Malaysian Agriculture Research and Development Institute. The stock-culture of DBM used throughout this study was reared on potted cabbage, *Brassica oleracea* var-*capitata*, in screen cages (38 cm×26 cm×26 cm) and maintained at 25°C ±5°C, with a photoperiod of 12 L: 12 D, and 60–80% relative humidity (RH). A 10% honey solution was offered as food to the adults, which had been reared over several generations in a laboratory before the experiments.

### Nosema sp. Spore Production

*Nosema* sp. spore suspensions were harvested from naturally infected DBM collected from a cabbage growing area in the Cameron Highlands, Pahang. No specific permits were required for the described field studies, and permission was provided by the landowners. However, the field studies did not involve endangered or protected species.DBM were homogenized in sterile water using a sterile mortar and pestle. The homogenate was partially purified by filtration through a nylon mesh cloth to remove tissue debris and centrifuged at 3000 rpm for 10 min. The supernatant was discarded, and the spore pellet was resuspended to 10 mL using sterile distilled water, and then the suspension was re-centrifuged. This procedure was repeated 3 times [5]. Next, 10 µL of the final spore solution was pipetted and poured into a hemocytometer for spore counting. The spores were counted under a light microscope at×40 magnifications using the Cantwell formula [6]. Spore suspensions, ranging from 1×10^2^ to 1×10^5^, were prepared by diluting with distilled water, and then stored at 4°C until further use.

### Pathogenic Effect of Nosema on *P. xylostella* Larvae

In all experiments, second- instar larvae were fed 5 mm wide leaf discs of rape, *Brassica juncea* plants that were treated with *Nosema sp.* spore suspension at concentrations (treatments) of 1×10^2^, 1×10^3^, 1×10^4^, and 1×10^5^ spores per larva. Sterile water was used as a control. Spore suspensions were spread evenly on the surface of the leaf discs using the bulb end of a Pasteur pipette. The larvae were selected randomly from the uninfected colonies and then placed in 24-well plastic culture plates, with one larva per well. The plates were placed in growth chambers that were maintained at 15, 20, 25, 30, or 35°C. All experiments were performed under similar RH (65% to 70%) and photoperiod (12∶12 h, light: dark) conditions. After 24 h, 10 randomly selected larvae were transferred to a plastic container (28 cm×16 cm×10 cm) containing moistened filter paper and untreated cabbage leaves, and the larvae were reared under different growth chamber conditions (15, 20, 25, 30, or 35°C, temperature; 65% to 70%, RH; and 12∶12 h, L:D). The cut end of the cabbage leaf was covered with water-soaked cotton wool in aluminum foil to maintain the freshness of the leaf. The lids of the containers were provided with nylon mesh-covered windows for ventilation. The treatments were replicated 5 times (n = 50). Food was renewed daily, and larval mortality was recorded every 24 h until all larvae had died or pupated. Healthy pupae were counted and placed individually in 2- cm-wide and 5–cm-deep small vials with cover. The newly emerged adults were used in the next experiments.

### Effect of Nosema on the Number of Emerged Adults and Egg Production of DBM

Adult males and females obtained from the study described in the previous section were counted. Twenty-four hours after adult eclosion, 2 females and 2 males from each treatment were placed in plastic containers (10 cm×10 cm×8 cm) containing a cabbage leaf for oviposition and a cotton ball soaked with 10% sugar solution as food. The leaves were checked daily for oviposition, and the number of eggs was recorded until no more eggs were laid. The experiments had a completely randomized design. The experiments were replicated 5 times spore concentration and per temperature. After the larvae hatched, 10 larvae per treatment were randomly sampled, placed in plastic containers (28 cm×16 cm×10 cm), and reared on fresh cabbage leaves until death or pupation. As soon as the larvae pupated, they were transferred into labeled 2- cm-wide and 5-cm-deep sterilized vials. The sex of each emerging adult was determined, and the emerged adults from each replicate were placed into plastic containers through a small hole on the cover and allowed to mate under laboratory conditions as previously described for the parents. This experiment was continued up to the fourth generation. The number of eggs produced and number of emerged adults for each treatment were recorded for 4 consecutive generations.

### Statistical Analysis

LC_50_ were analyzed at 24, 48, and 72 h after treatment using probit analysis [7], and calculated LC_50_ values were considered to be significant if their 95% confidence intervals did not overlap. Statistical software POLO-PC was used for LC_50_ analysis. Percentage mortality was subjected to analysis of variance (ANOVA), and means were compared using Tukey’s test, with a reference probability of *p*≤0.05; analyses were run on MiniTab software version 16. The effects of temperature, *Nosema* spore concentration, and generation number, as well as their interactions, on the number of eggs produced and number of emerged adults were evaluated separately for each treatment by ANOVA using the General Linear Model. When values of ANOVA were significant, means were separated using Tukey’s range test at *p* = 0.05.

### Histological Preparations

In this study, a 1×10^3^ spore concentration was chosen to investigate the effect of *Nosema* on the midgut of DBM larvae because it was the intermediate concentration in our experiment. In general, late second-instar larvae were allowed to ingest 10^3^ spores placed on 5 mm cabbage leaf discs. A larva was placed in each well of 24-well plastic culture plates. Larvae were maintained on the *Nosema* contaminated discs for 24 h. Larvae that did not consume the entire disc during this period were removed from the experiment. After larvae ingested the leaf pieces, they were transferred to new plastic containers (10 cm×10 cm×8 cm) containing fresh cabbage leaves and then dissected at 24, 48, and 72 h post-treatment, fixed in Bouin’s solution for 24 h, dehydrated in an ethanol series, and embedded in paraffin wax (58°C to 60°C). Sections were cut using a rotary microtome (5 µm thickness) and stained with hematoxylin-eosin [8]. After staining, sections were dehydrated, cleared in xylene, and mounted in Canada balsam. Mounted sections were examined using an Olympus BX43 light microscope equipped with an Olympus DP72 10 megapixel camera.

## Results

### Pathogenicity of Nosema to Larval Stage of P. xylostella

The susceptibility of the second-instar larvae to *Nosema* was tested by leaf dip bioassay, in which the larvae were allowed to feed on leaf discs treated with 1×10^2^, 1×10^3^, 1×10^4^, and 1×10^5^ spore concentrations at 24, 48, and 72 h post-treatment (Table 1). The results showed that this insect was highly susceptible at 24 h after treatment with *Nosema* at 35°C [50% lethal concentration (LC_50_), 32652 (spore/µL) of *Nosema*] (Table 1). The toxicities of *Nosema* at 30, 25, 20, and 15°C were 19, 86, 58, and 32 times lower than that at 35°C, respectively. The same trend was observed for the second-instar larvae at 48 and 72 h. However, the LC_50_ [474.2 (spores/µL)] for DBM after 72 h at 35°C was the lowest recorded in this study. Overall, the LC_50_ values declined with increasing temperature and durations of exposure to *Nosema* (length of bioassay).

**Table 1:** Results of probit analysis on the effect of *Nosema sp*. infection on *Plutella xylostella* LC50 stands for Lethal Dose 50 (concentration in water having 50% chance of causing death); F symbolizes F-statistic; d.f. stands for degrees of freedom.

Temperature	Statistical Data(probit analysis)	Time after infection		
		24 hours	48 hours	72 hours
15°C	LC_50_(spore/*µl*)^a^	0.106×10^7^	0.676×10^6^	0.427×10^6^
	95% CL	0.189×10^6^–0.833×10^8^	0.123×10^6^–0.786×10^8^	81059–0.669×10^8^
	Slope ± SE	0.504±0.122	0.469±0.125	0.451±0.130
	Chi- square(d.f.)	0.74(2)	0.44(2)	0.73(2)
20°C	LC_50_(spore/*µl*)^a^	0.189×10^7^	0.356×10^6^	0.156×10^6^
	95% CL	0.200×10^6^–0.185×10^10^	70895–0.291×10^8^	35789–0.759×10^7^
	Slope ± SE	0.373±0.103	0.432±0.116	0.420±0.116
	Chi- square(d.f.)	0.64(2)	0.17(2)	0.21(2)
25°C	LC_50_(spore/*µl*)^a^	0.283×10^7^	0.135×10^7^	0.124×10^7^
	95% CL	0.376×10^6^–0.399×10^10^	0.241×10^6^–0.149×10^9^	0.164×10^6^–0.180×10^10^
	Slope ± SE	0.605±0.183	0.568±0.145	0.445±0.137
	Chi- square(d.f.)	0.31(2)	1.25(2)	0.32(2)
30°C	LC_50_(spore/*µl*)^a^	0.642×10^6^	0.486×10^6^	0.380×10^6^
	95% CL	0.133×10^6^–0.272×10^8^	72823–0.246×10^9^	70814–0.101×10^9^
	Slope ± SE	0.497±0.115	0.378±0.114	0.443±0.136
	Chi- square(d.f.)	0.39(2)	0.27(2)	0.19(2)
35°C	LC_50_(spore/*µl*)^a^	32652	858.757	474.216
	95% CL	7914.8–0.728×10^6^	93.502–3053.507	31.005–1761.598
	Slope ± SE	0.345±0.098	0.399±0.104	0.431±0.115
	Chi- square(d.f.)	0.89(2)	0.07(2)	0.36(2)

a n = 250.

**Figure 1:**
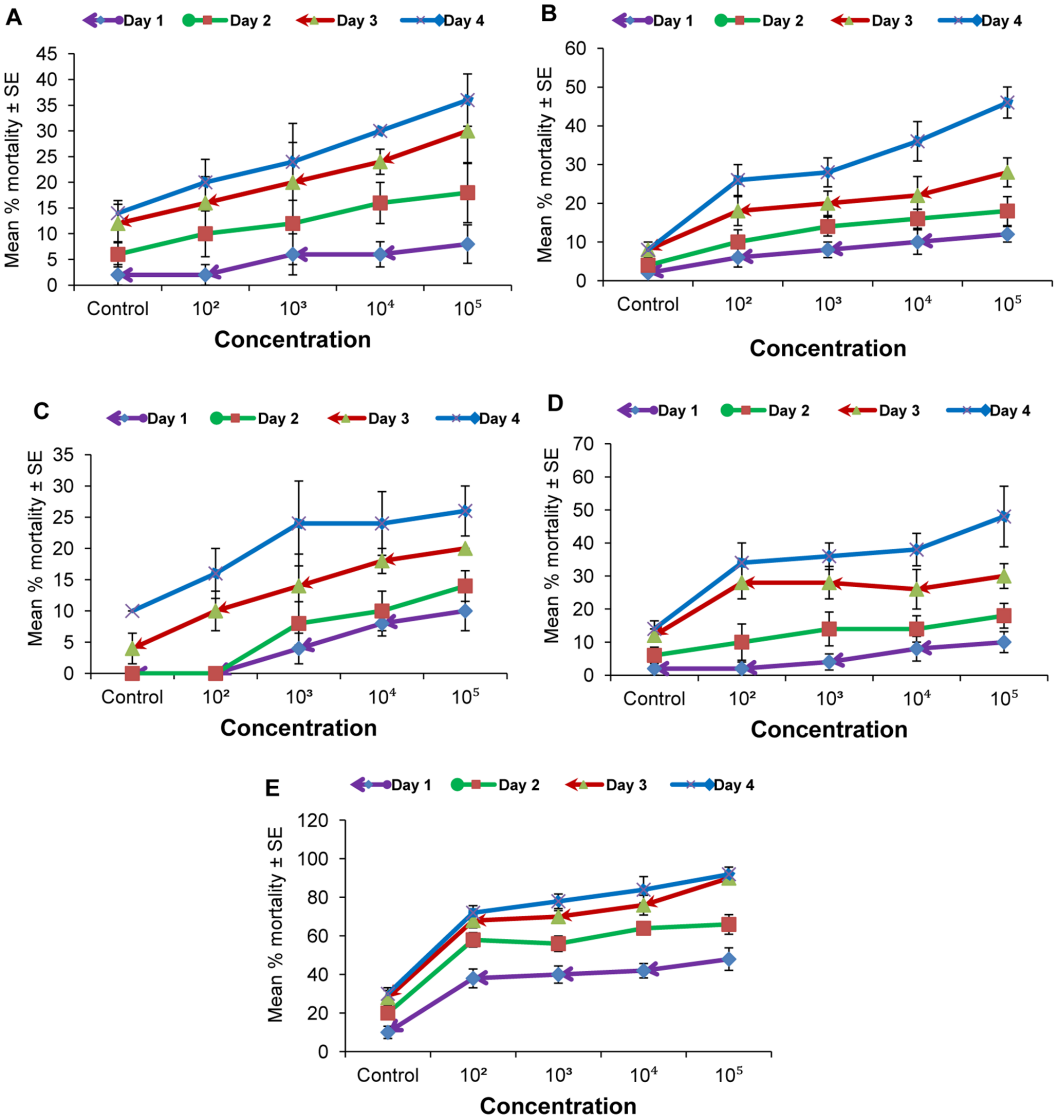
Mortality induced by *Nosema* sp. Mean percent (accumulative) mortality (SE) of diamondback moth larvae fed rape (*Brassica juncea*) leaves treated at various concentrations of *Nosema* spores; 15° (A), 20° (B), 25° (C), 30° (D) and 35°C (E) in leaf dip bioassay. Vertical bars = S.E.

The results of the laboratory bioassay (leaf dip) showed that all *Nosema* concentrations tested at different temperatures against the second instar larvae of *P. xylostella* caused various levels of mortality at all temperatures (Figure 1). As expected, the mortality of the second- instar DBM fed with *Nosema-*treated discs significantly increased with increasing spore concentrations (Figure 1), where the greatest accumulated larval mortality (92%) was observed when 1×10^5^ spores were applied at 35°C (F = 93.82, d.f. = 4, *P* = 0.0; Figure 1E), and subsequently at 20 C (Figure 1B) and 30°C (≃50% Figure 1D), with the lowest mortality (26%) observed at 25°C (Figure 1C).

The larval mortality was also significantly affected by the time (days) after spore ingestion at 15°C (F = 17.85, d.f. = 3, *p*<0.05), 20°C (F = 42.50, d.f. = 3, *p*<0.05), 25°C (F = 25.09, d.f. = 3, *p*<0.05), 30°C (F = 37.83, d.f. = 3, *p*<0.05), and 35°C [F = 73.66, d.f. = 3, *p*<0.05 (Figure 1)]. However, no significant interaction (*p*>0.05) occurred between concentration and time (days) for all temperatures in terms of influencing the larval mortality.

### Effect of Nosema on the Number of Emerged Adults and Egg Production

**Figure 2:**
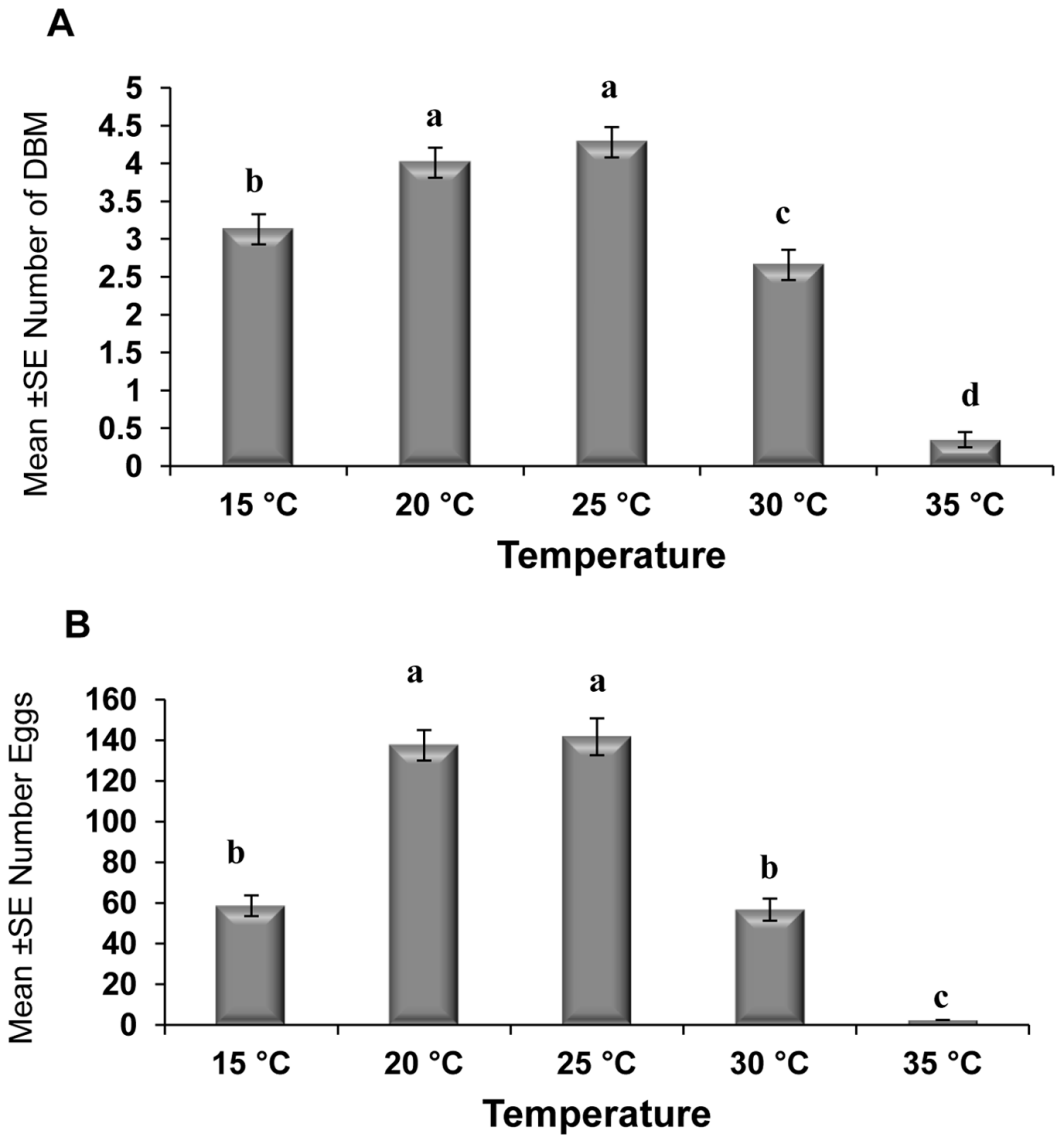
Effect of temperature on adult emergence and egg production. Graph bars represent mean ± SE of number of adults emerged (a), number of eggs produced by DBM adults (b) developed from *Nosema* infected DBM larvae at 15°, 20°, 25°, 30° and 35°C. Different letters above error bars indicate significant difference (P<0.05).

**Figure 3:**
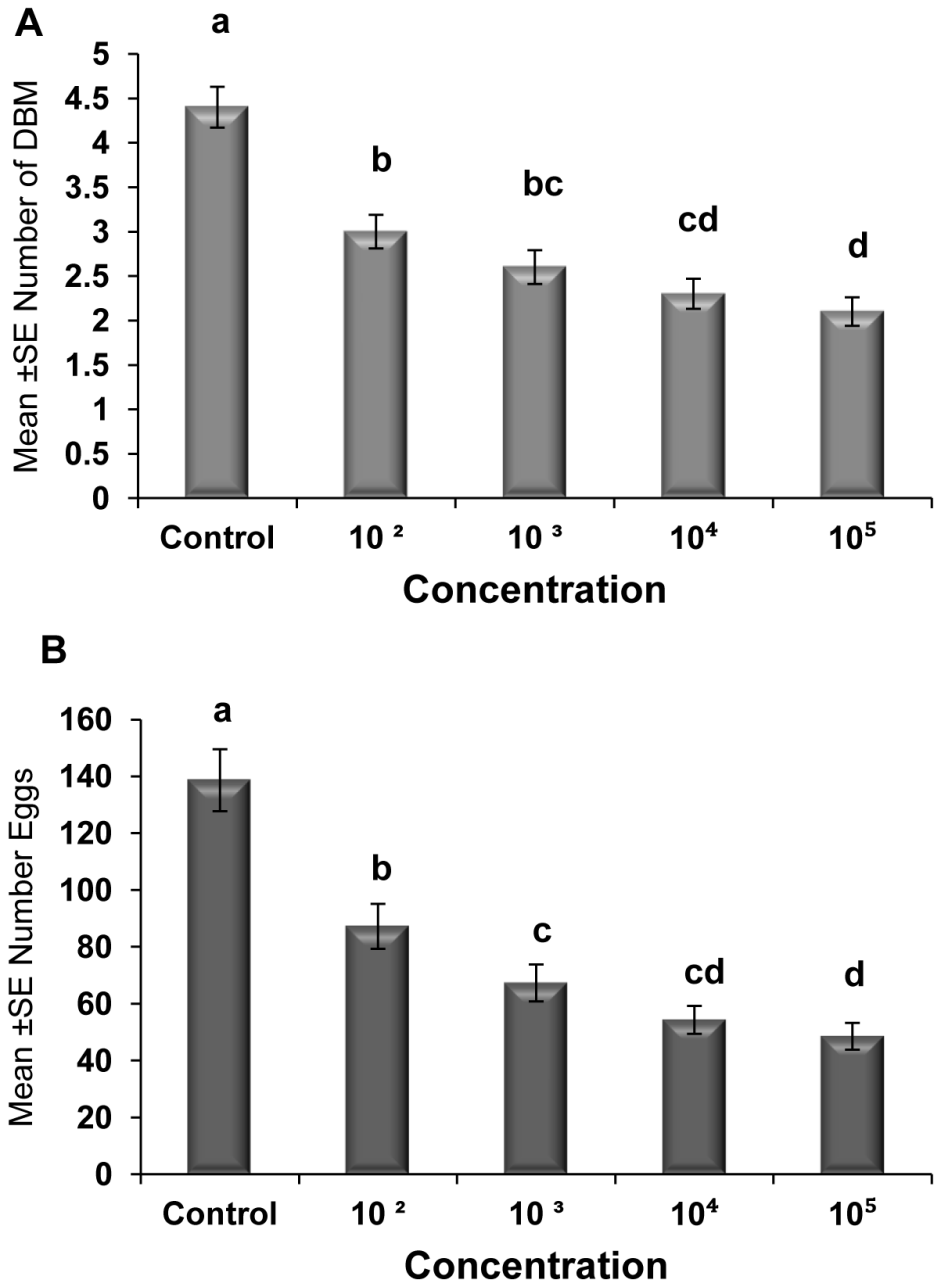
Effect of concentration of *Nosema* on adult emergence and egg production. Graph bars represent mean ± SE of number of adults emerged (a), number of eggs produced by DBM adults (b) developed from larvae inoculated at different dosages of *Nosema* sp. spores. Different letters above error bars indicate significant difference (P<0.05).

**Figure 4:**
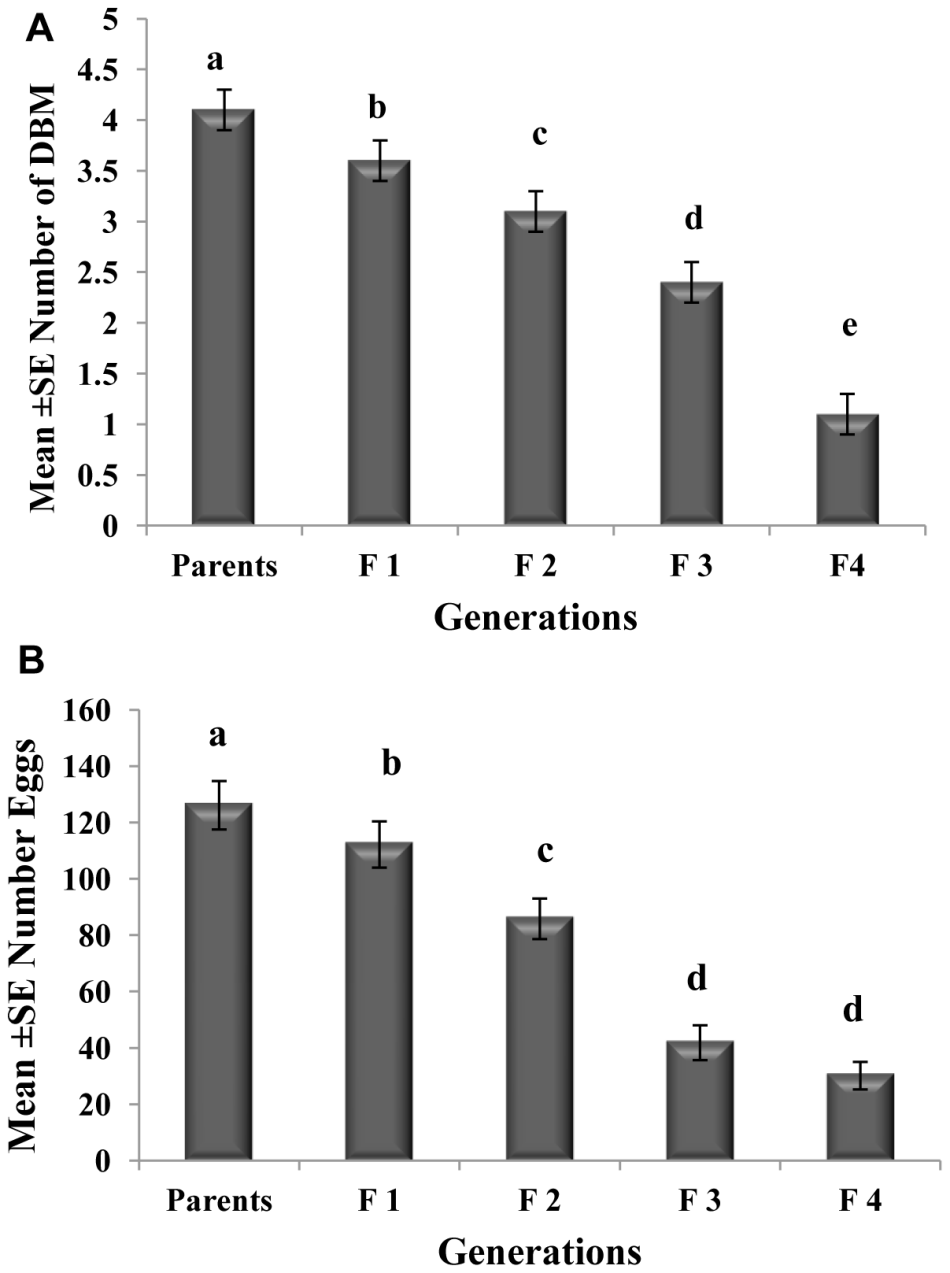
Comparison of adult emergence and egg production through successive generations. Graph bars represent mean ± SE of number of adults emerged (a), number of eggs produced by DBM adults (b) developed from *Nosema* infected DBM larvae through successive generations. Different letters above error bars indicate significant difference (P<0.05).

**Figure 5:**
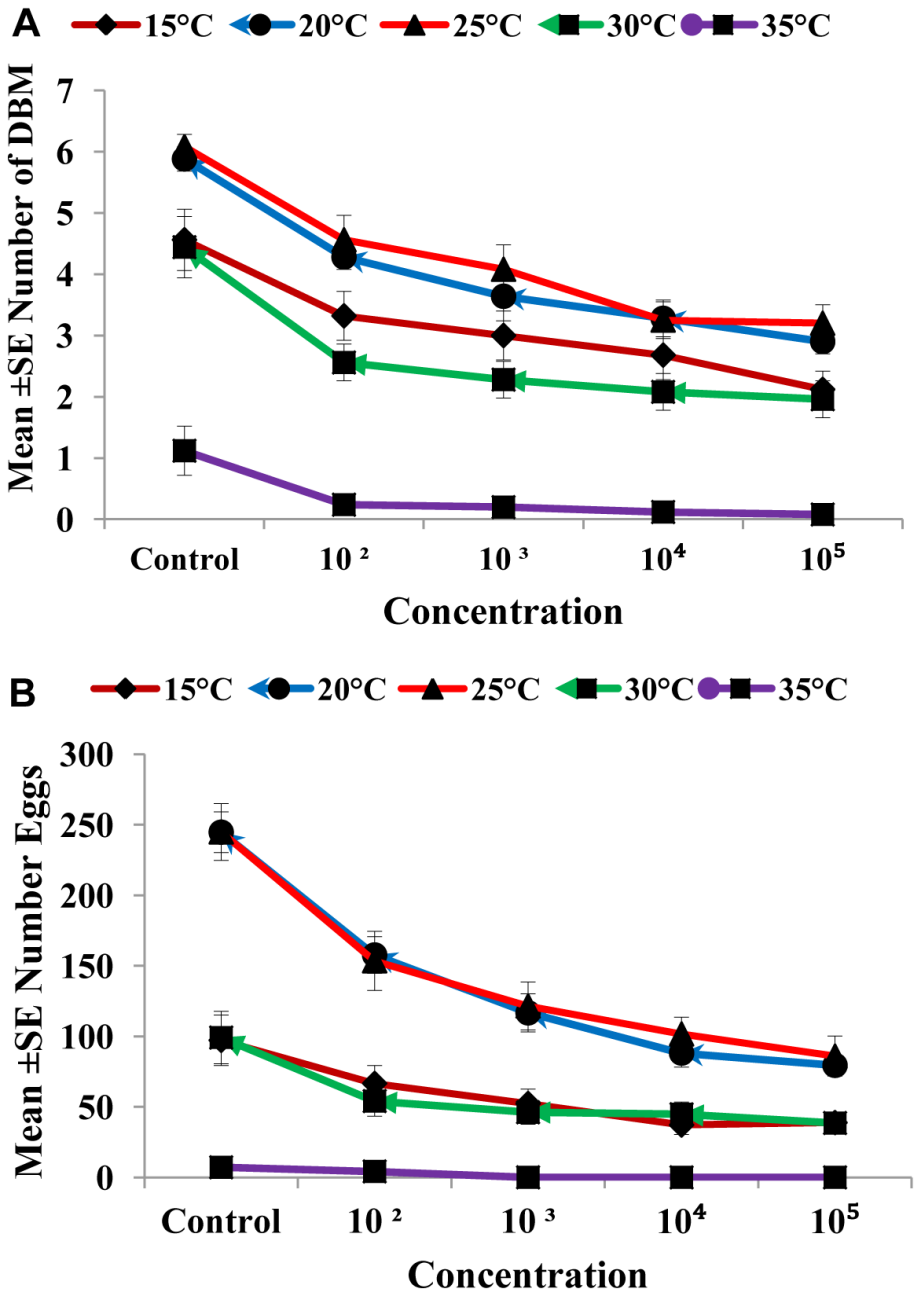
Influence of *Nosema* concentration and different temperatures on adult emergence and egg production. Data represent the mean ± SE of number of adults emerged (a), number of eggs produced by DBM adults (b) developed from *Nosema* infected DBM larvae at 15°, 20°, 25°, 30° and 35°C. Vertical bars = S.E.

**Figure 6:**
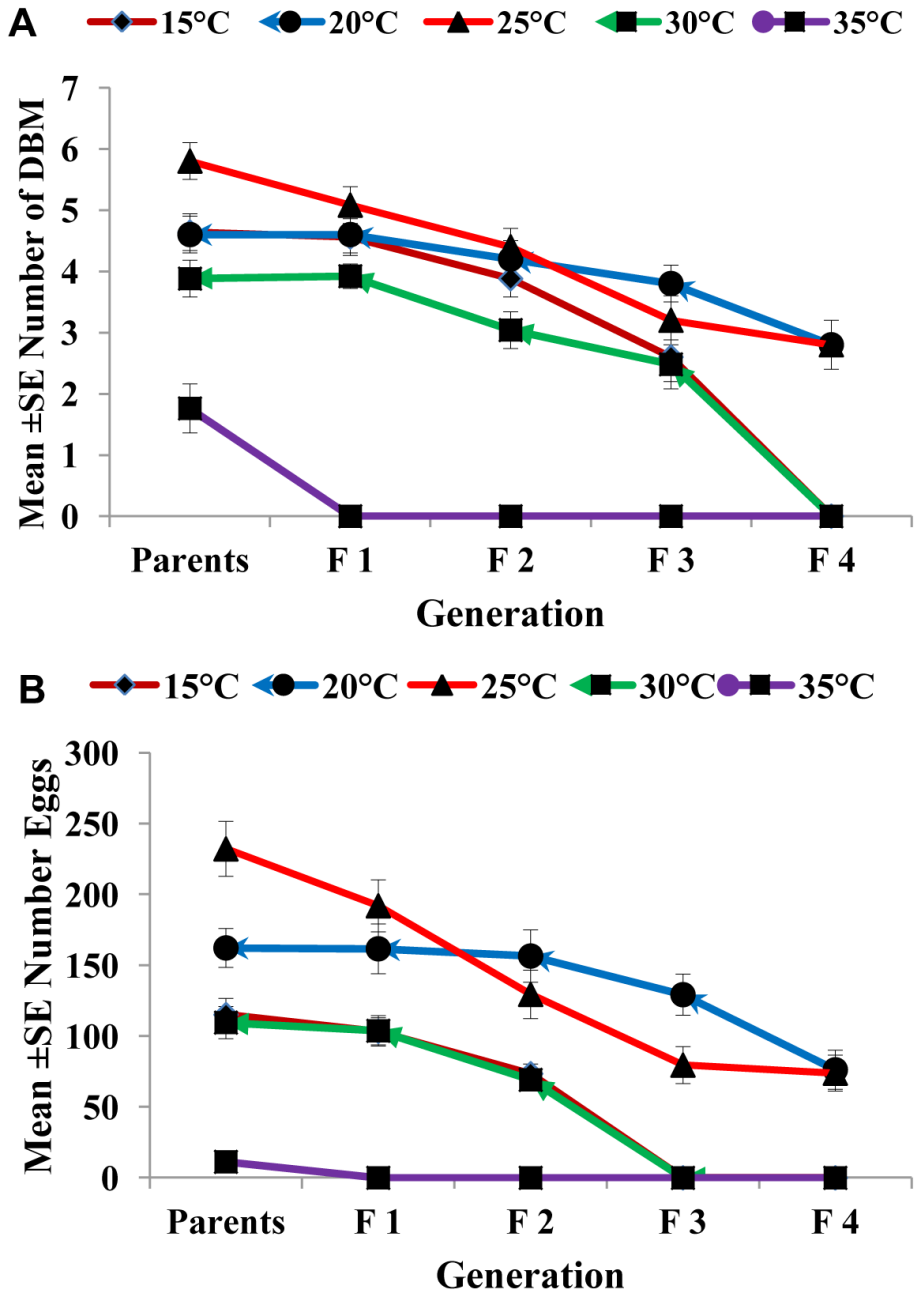
Influence of temperature on adult emergence and egg production of *P. xylostella* through successive generations. Data represent the mean ± SE of the number of adults emerged (a), number of eggs produced by DBM adults (b) developed from *Nosema* infected DBM larvae through successive generations. Vertical bars = S.E.

The effects of different spore concentrations of *Nosema* on the number of emerged adults and eggs produced by DBM adults developed from infected larvae at 15, 20, 25, 30, and 35°C are shown in Tables 2 and 3. The results indicated that all the main effects (temperature, concentration, and generation) and the respective interaction terms were highly significant for both mean numbers of DBM adults and eggs produced (Tables 2 and 3), except the interaction between concentration and generation for emerged adults and the interaction between temperature, concentration and generation for eggs produced. The highest reductions in the number of DBM were most pronounced at temperatures of 35 and 30°C (Figure 2a), and the highest reduction in the number of eggs was noted at 35°C (Figure 2b). The largest reduction in the number of emerged adults and number of eggs laid by infected moths were observed in the larvae exposed to 1×10^5^ spores/larvae. Other treatments also significantly reduced the number of emerged adults and eggs laid (1×10^2^, 1×10^3^, and1×10^4^) compared with that of the control (Figures 3a and b). The reduction in the number of eggs laid and adults emerged was also noted in the subsequent generations, particularly the third and fourth generations (Figures 4a and b). In general, the number of emerged adults and egg produced showed inverse relationships with spore concentrations at all temperatures (Figure 5a and b). Temperature significantly affected the number of emerged adult and egg produced at all concentrations. However, the lowest numbers were observed at 35°C at all concentrations compared with those at other temperatures. Interestingly, at 35°C, the adults failed to emerge both at low and high doses of *Nosema* spores, and the number of eggs produced were adversely affected (Figures 5a and b). Progeny production through generations was also significantly (*p*<0.05) affected by *Nosema* infection. The production of progeny was markedly affected as the generation number increased (Figures 6a and b). The population size was collapsed after the first and third generations at 35°C and at 15 and 30°C, respectively.

**Table 2:** Result of ANOVA for adults emerged as infected by *Nosema* infection at various spore concentrations, temperatures through five generations.

Effect	Df	*F*	*P- va*lue
Temperature	4	267.1	<0.05
Concentration	4	92.8	<0.05
Generation	4	148.6	<0.05
Temperature×Concentration	16	2.34	<0.05
Temperature×Generation	16	12.02	<0.05
Concentration×Generation	16	1.45	>0.05
Temperature×Concentration×Generation	64	2.35	<0.05
Error	500		

**Table 3:** Result of ANOVA for eggs produced by adults infected with *Nosema* at various spore concentrations, temperatures through five generations.

Effect	Df	*F*	*P- va*lue
Temperature	4	293.2	<0.05
Concentration	4	109.9	<0.05
Generation	4	148.6	<0.05
Temperature×Concentration	16	12.9	<0.05
Temperature×Generation	16	13.5	<0.05
Concentration×Generation	16	2.61	<0.05
Temperature×Concentration×Generation	64	1.01	>0.05
Error	500		

### Effect of Nosema on the Histopathology of midgut of *P. xylostella* Larvae

**Figure 7:**
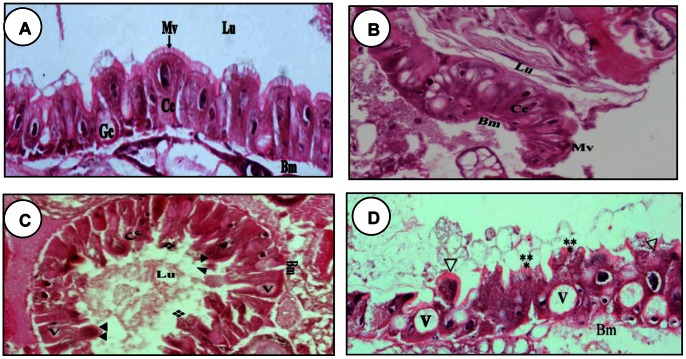
Histopathological effects of *Nosema* in on *P. xylostella* midgut. General aspects of the midgut larvae (A) and histopathological effects of *Nosema* on it (B) after 42 h of treatment, (C) after 48 h of treatment, (D) after 72 h of treatment. In C&D arrows indicate lysis of columnar cells and disrupted columnar cells (stars) are sloughed into the lumen of midgut. V, vacuoles; Lu, lumen; Gc, goblet cell, Cc, columnar cell; Mv, microvilli; Bm, basement membrane. Magnification 40 x.

The midgut of *P. xylostella* larvae is a long straight tube, which consists of columnar and goblet cells linked by well-developed border of microvilli. The epithelial cells rest on a basement membrane and muscle fibers (Figure 7A). Sections from larvae that had been fed for 24 h with *Nosema-*infected leaves showed distended and bulbous distal ends of the epithelium columnar cells (Figure 7B). Although the goblet cells may show some morphological changes, there were no signs of damage at this stage (Figure 7B). Degeneration of the epithelium columnar cells continued, such that after 48 h of ingestion of a *Nosema-*infected food, the lumen exhibited debris of disrupted cells (Figure 7C). The goblet cells also showed signs of damage after 48 h, but both types of cells were still attached to the basement membrane. At 72 h post-treatment, the epithelial cells exhibited swelling, and some microvilli were disrupted because of the swelling of the cells and lysis of cytoplasmic material (Figure 7D). In addition, the vacuoles increased in size, and some columnar cells were dislodged and sloughed into the lumen of the midgut (Figure 7D, arrows). This result clearly showed that *Nosema* infection had caused cytopathological changes in the midgut epithelial cells.

## Discussion

The present results showed that *Nosema* is highly pathogenic to *P. xylostella (* Table 1), confirming previously reported data that DBM larval mortality was high even at low concentrations of *Nosema* spores [4] and for the gypsy moth (*Lymantria dispar* L.), where mortality varied between 79% and 99%, independently of the spore concentration [9]. The silkworm has also been reported to show high larvae mortality upon infection by *Nosema* sp [10], [11]. In contrast, minimal mortality (3%) was observed in southwestern corn borer *Diatraea grandiosella* larvae infected with *Nosema* sp. (isolate 506), even at high doses [12].

Our results showed that the susceptibility of the *P. xylostella* larvae to the pathogen was dose-dependent. More than 90% of larvae died by day 4 after treatment at a dose (spore concentration) of 10^5^ (spores/µL) compared with those at lower doses. The mortality of *P. xylostella* larvae at higher doses might be attributed to intestinal damage and bacterial septicemia as a result of *Nosema* infection [13]. Temperature had a negative effect on mortality, where the greatest accumulated larval mortality (92%) was observed at 35°C, followed by at 20 and 30°C (≃50%), with the lowest accumulated mortality (26%) observed at 25°C, which was considered to be the optimum temperature. The mortality in *Nosema pyrausta-*infected *Ostrinia nubilalis* larvae was reported to be high at 24°C than 30°C [14]. This suggests that larvae are able to survive longer at low temperatures than at higher temperatures. An increase in mortality over days could have resulted from the increasing damage that spores caused while spreading in the body of the larvae. This eventually led to tissue destruction, and hence the larvae died before pupation or even if they reached the pupation stage, the number of adults emerged is less and their fecundity is low. However, the larvae were unable to bear the impact of the interaction between high temperature and *Nosema* infection, and mortality continued to increases over time, indicating an additive effect of *Nosema* infection, particularly at 30 and 35°C. Therefore, there was high mortality reaching a peak (92%) at 35°C. *Nosema* sp. spores were found in the midgut of *Antheraea mylitta* at the beginning of infection, and then spread to the fat body, and infection was observed in the tracheal epithelium, Malphigian tubules, and gonads during the later stages [15].

These results suggest a clear impact of both *Nosema* infection and temperature on *P. xylostella* egg production and number of adults emerging. The reduction in the number of egg production may be related to several factors affecting the egg laying process, because *Nosema* infects certain tissues involved in egg production [16]. The high spore concentration of *Nosema* in the gonads of *A. mylitta, Antheraea assamensis*, and *Bombyx mori* was found to affect reproductive potential and fertility [17]. Reduction in the number of eggs deposited by infected females could be a response to the competition between the host and the microsporidia for nutrients [18], [19]. In addition, infected females have been suggested to compensate for the loss of nutrients to the microsporidia by producing fewer eggs [19], [20]. Our observations are consistent with the effects of other *Nosema* spp., such as *N. pyrausta* on the European corn borer, *Ostrinia nubilalis* (Hübner) [21], and *Nosema* sp. on silkworm [10], [22].

Our results also indicate that the impact of both *Nosema* infection and temperature on the number of adults emerging from infected larvae occurred over 4 subsequent generations. This reduction in the number of the progeny could have been caused by the larval and pupae mortality as a result of the damage and changes in the organs of the *Nosema-*infected insects. This effect is passed on through generations, leading to fewer healthy adults and, eventually, population collapse. These findings are in accordance with previous results that indicate similar sub-lethal effects of other microsporidia on insects [23]. For example, the effectiveness of the microsporidium *Edhazardia aedis* (Kudo) in controlling a semi-natural population of *Aedes aegypti* (L.) was evaluated over a 2-year period, which led to the successful elimination of a population of *A. aegypti* in Florida [24].

In general, low and high temperatures had adverse effects on the production of progeny of the infected DBM, particularly at 35°C where the progeny failed to emerge. However, high temperature is not as stable in natural conditions as it is under laboratory conditions; temperature fluctuates within a day, and it is usually cooler at night than in the day. Thus, future studies will be aimed toward the development of an efficient field trial before the possible commercial production of *Nosema* as a biocontrol agent of DBM in the field.

Since the midgut performs important functions such as digestion and absorption, we presume that the negative effects of *Nosema* infection on DBM was partially because of the damage it causes to the midgut epithelium, leading to digestive and food absorption disorders. Subsequently, vacuolization occurred as a result of cell elongation after infection [25]. The disruption of the microvilli and vacuolization of the intracellular organelles in the midgut epithelial cells were similar to the cytotoxic effects observed in the midgut of *P. xylostella* after treatment with *Bacillus thuringiensis* [26] and *Vairimorpha* sp. [27]. All these changes have been reported in several susceptible Lepidoptera [28].

The histopathological changes caused by *Nosema* infection suggest that Nosema sp. could be fatal to DBM if infected with a sufficient amount of spores. In conclusion, the result of the present study suggests that *Nosema* infection has a highly negative impact on DBM, and thus, its utilization could be considered in IPM. However, more studies are still required in this regard.
