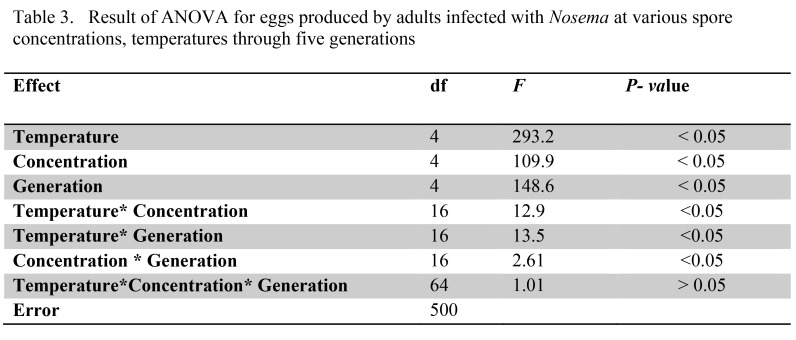# Correction: Pathogenicity of *Nosema* sp. (Microsporidia) in the Diamondback Moth, *Plutella xylostella* (Lepidoptera: Plutellidae)

**DOI:** 10.1371/annotation/4ba70a5b-bfd5-4335-9f1b-dd96afd9a6d5

**Published:** 2014-01-17

**Authors:** Nadia Kermani, Zainal-Abidin Abu-hassan, Hamady Dieng, Noor Farehan Ismail, Mansour Attia, Idris Abd Ghani

Formatting errors were introduced into tables 2 and 3 during the preparation of this article for publication. Please view the correct tables here:

Table 1: 

**Figure:**
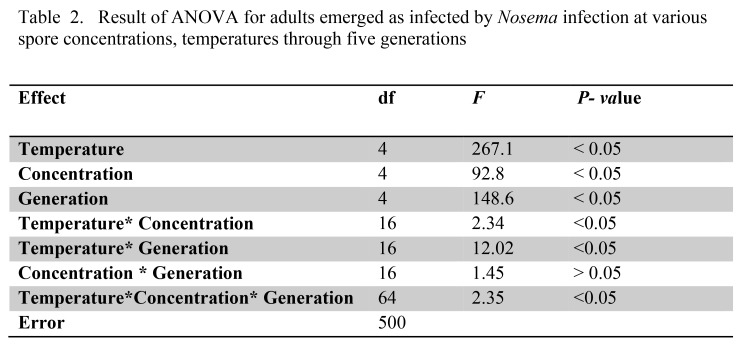


Table 2: 

**Figure:**